# AI-Assisted Identification of the Medial Lingual Foramen on CBCT: A Deep Learning Approach for Preoperative Implant Assessment

**DOI:** 10.3390/medicina62061059

**Published:** 2026-05-30

**Authors:** Alina Ban, Sorana Mureşanu, Raluca Roman, Liviu Iacob, Mihaela Hedeşiu, Cristian Dinu, Oana Almăşan, on behalf of Team Project Group

**Affiliations:** 1 Department of Oral and Maxillofacial Surgery and Radiology, Iuliu Hațieganu University of Medicine and Pharmacy, 32 Clinicilor Street, 400006 Cluj-Napoca, Romania; 2 Department of Computer Science, Technical University of Cluj-Napoca, 400114 Cluj-Napoca, Romania; 3 Department of Prosthetic Dentistry and Dental Materials, Iuliu Hațieganu University of Medicine and Pharmacy, 400006 Cluj-Napoca, Romania

**Keywords:** cone-beam computed tomography, medial lingual foramen, deep learning, image segmentation, implant dentistry, artificial intelligence, risk assessment

## Abstract

*Background and Objectives*: Although the anterior mandible is generally considered a safe region for implant placement, injury to the medial lingual foramen (MLF) may result in significant vascular complications. Accurate identification of this structure is challenging due to its small size, low volumetric representation, and anatomical variability. This study aimed to evaluate the anatomical characteristics of the MLF using cone-beam computed tomography (CBCT) and to develop and validate a deep learning-based approach for its automated detection and segmentation. *Materials and Methods*: A total of 106 CBCT scans were retrospectively analyzed to assess the morphology and position of the MLF. Manual pixel-wise annotations of the complete canal trajectory were performed on sagittal slices and used to train convolutional neural network models based on a U-Net-derived framework. Multiple configurations, including multi-class, binary, two-dimensional, and three-dimensional approaches, were evaluated. Given the extremely limited volumetric representation of the MLF, severe class imbalance represented a major challenge during model training and evaluation. Model performance was assessed using the Dice similarity coefficient, precision, recall, and Hausdorff distance. External validation was performed on an independent dataset of 10 CBCT scans. *Results*: The MLF was identified in all patients, with a single canal observed in 63% of cases. The sagittal-plane binary segmentation model achieved the best performance, with a test Dice score of 0.79, precision of 0.88, and recall of 0.73. External validation demonstrated a Dice score of 0.81, precision of 0.89, and recall of 0.71. The 95th percentile Hausdorff distance was 2.6 mm, and the mean center-point localization error was 1.2 mm. The model correctly detected the MLF in 90% of external cases. *Conclusions*: Deep learning-based segmentation of the MLF is feasible and may support automated localization assistance during preoperative CBCT assessment. Performance was influenced by the alignment between the annotation strategy and model input, highlighting an important consideration for small-structure segmentation. Further validation on larger multicenter datasets is required before clinical implementation can be considered.

## 1. Introduction

The mandibular symphyseal region has traditionally been considered a surgically safe zone for implant placement. However, increasing clinical evidence has demonstrated that vascular injury to the lingual foramen during implant surgery may result in significant complications, including hemorrhage, hematoma formation, and in severe cases, airway obstruction due to elevation of the floor of the mouth and tongue [1,2]. Additionally, cases of transient or permanent neurosensory disturbances following implant placement in the anterior mandible have been reported [3]. These findings highlight the necessity of accurate preoperative identification of the mandibular lingual foramen to minimize surgical risks [4].

Anatomical studies describe the lingual foramina as small vascular openings located on the lingual surface of the mandible [5]. Based on their position, they are classified as medial lingual foramina (MLF), located near the mandibular midline, and lateral lingual foramina positioned more posteriorly [6]. The MLF is particularly relevant in implantology due to its proximity to common implant insertion sites in the interforaminal region. However, considerable variability in number, diameter, and spatial relationship to anatomical landmarks complicates clinical risk assessment [7].

Cone-beam computed tomography (CBCT) has become the imaging modality of choice for preoperative implant planning, providing high-resolution three-dimensional (3D) visualization and accurate morphometric assessment of maxillofacial structures [8]. Despite these advantages, CBCT interpretation remains operator dependent, and the identification of small vascular foramina may be challenging, leading to potential underestimation of surgical risk [9].

Artificial intelligence (AI) has increasingly been used in dental imaging for automated detection and segmentation of anatomical structures, improving diagnostic consistency and efficiency [10,11]. Nevertheless, the use of AI for precise identification and segmentation of the MLF has not been sufficiently investigated.

The proposed framework is intended as a decision-support and automated localization-assistance tool that may facilitate the identification of anatomically high-risk regions during preoperative implant assessment. Therefore, the aims of this study were (i) to evaluate the anatomical characteristics and position of the MLF using CBCT imaging and (ii) to develop and validate an AI-driven tool for accurate segmentation and detection of the MLF. By integrating morphometric CBCT analysis with automated AI-based identification, this study seeks to enhance reproducibility and standardization in preoperative implant assessment. The primary endpoint for the AI component was segmentation performance assessed using the Dice similarity coefficient and case-level localization accuracy.

## 2. Materials and Methods

This retrospective study included 106 CBCT scans obtained from patients who required oral surgical interventions at the Department of Maxillo-Facial Surgery, University of Medicine and Pharmacy in Cluj-Napoca, Romania. The study protocol complied with ethical standards and was approved by the Research Ethics Committee of the University of Medicine and Pharmacy Cluj Napoca, Romania, 326/01.10.2021. All images were anonymized prior to analysis.

All scans were acquired using a Planmeca ProMax 3D Classic unit (Helsinki, Finland) following a standardized acquisition protocol: 90 kilovoltage (kV), 10 milliamperage (mA), 15 s exposure time, field of view (FOV) 80 × 50 mm and voxel size 0.2–0.3 mm. Patients were positioned in a natural head position for all examinations.

Inclusion criteria consisted of patients aged ≥ 18 years with high-quality CBCT scans of the mandible and no evidence of mandibular pathology or prior surgical intervention in the anterior region.

Exclusion criteria included low-quality scans with artifacts, incomplete visualization of the inferior mandibular border, history of trauma or surgery in the anterior mandible, edentulous patients, and age under 18 years.

Manual annotations and morphometric evaluations were performed independently by two experienced and calibrated investigators (A.B., S.M.) using Planmeca Romexis^®^ software (Version 6.4) (Helsinki, Finland). In cases of disagreement, a third experienced evaluator (M.H.) was consulted, and consensus was reached.

A standardized measurement protocol was applied. The following parameters were recorded: patient age and sex, number of MLFs (single, double, triple), canal height (LH), width (LW), length (LL), and direction (ascending or descending). Width measurements were performed on axial CBCT sections, whereas all other measurements were obtained from sagittal reconstructions. Canal height was defined as the mean of three measurements taken along the canal trajectory.

**Figure 1:**
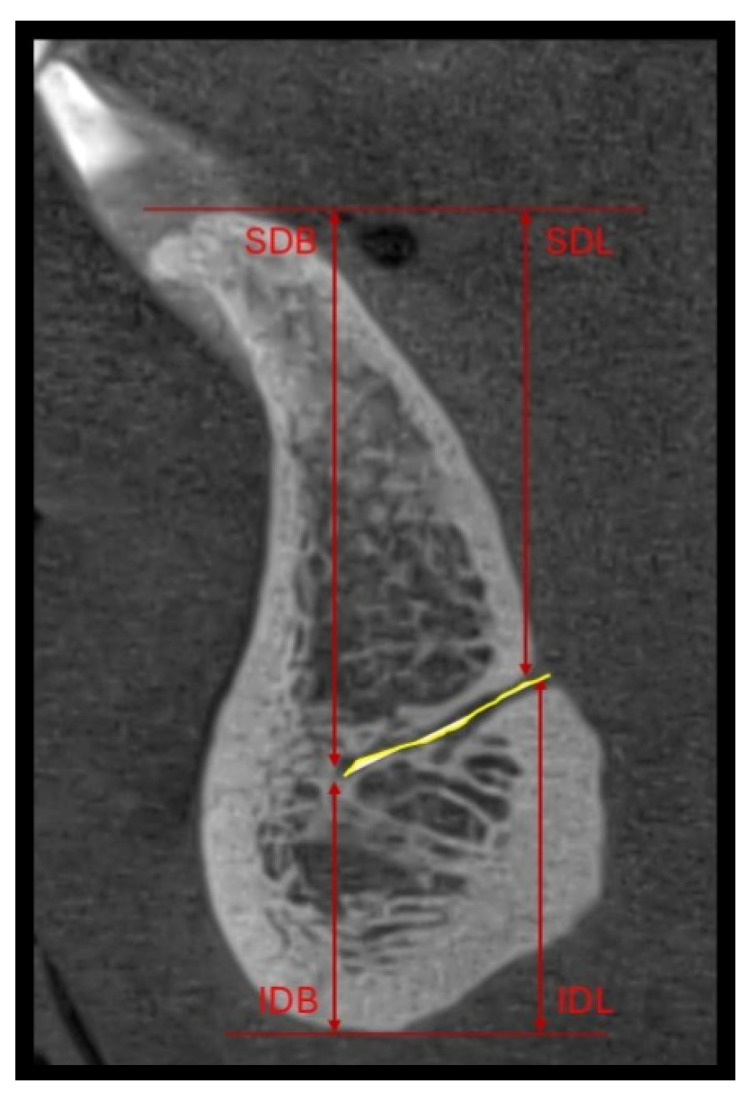
CBCT sagittal cut showing study variable measurements: superior buccal—superior border to the buccal terminal (SDB); superior lingual—superior border to the lingual terminal (SDL); inferior buccal—inferior border to the buccal terminal (IDB), inferior lingual—inferior border to the lingual terminal (IDL); Yellow line—lingual canal.

**Figure 2:**
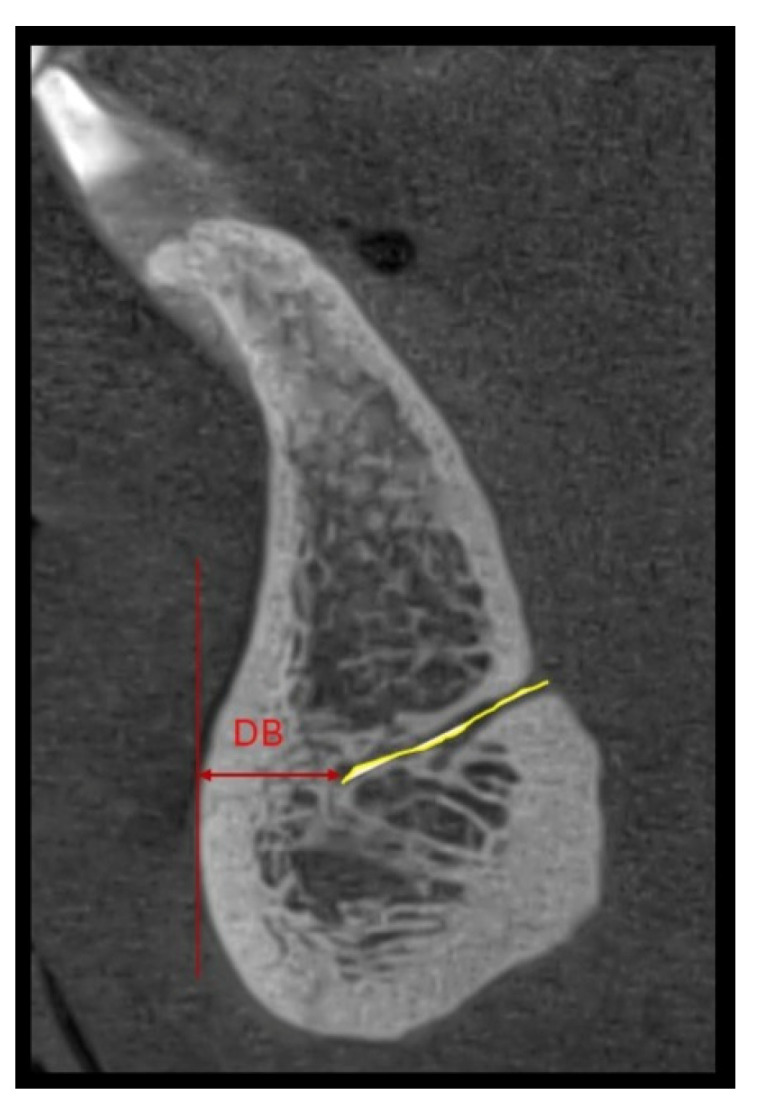
CBCT sagittal cut showing the deepest buccal distance of the MLF (DB). Yellow line—lingual canal.

Distances to cortical surfaces were also assessed, including superior buccal (SDB), superior lingual (SDL), inferior buccal (IDB), inferior lingual (IDL) (Figure 1), and the deepest buccal distance of the MLF (DB) (Figure 2). In cases with multiple canals, measurements were performed independently for each canal.

Interobserver agreement for categorical variables (presence and number of MLF) was assessed using Cohen’s kappa coefficient, while the reliability of continuous morphometric measurements was evaluated using the intraclass correlation coefficient (ICC). Values > 0.75 were considered indicative of good agreement.

### Deep Learning Model Development

The dataset consisted of CBCT scans from 106 patients stored in the original 16-bit DICOM format. Seven patients were randomly selected and reserved as an independent test cohort. The independent test cohort was excluded prior to cross-validation and was not used during model training or hyperparameter optimization. All volumes exhibited isotropic voxel spacing and equal slice numbers across axial, sagittal, and coronal planes. All CBCT volumes were resampled to isotropic voxel spacing and intensity-normalized prior to training. Automatic cropping was applied to focus on the mandibular region. The nnU-Net framework automatically applied preprocessing and augmentation procedures, including random rotations, scaling, mirroring, and intensity variations. Training was performed using stochastic gradient descent with adaptive learning rate scheduling, as configured by the nnU-Net framework. Batch size, patch size, and optimization parameters were automatically selected based on dataset characteristics.

Manual annotations were performed in 3D Slicer (v4.13.0) on sagittal slices. Annotations were performed using standardized CBCT visualization settings, with image magnification adjusted to optimize visualization of the MLF. All annotations were reviewed and validated by an experienced dentomaxillofacial radiologist (M.H.). Annotations were performed as pixel-wise segmentations of the complete visible canal trajectory rather than isolated foraminal entry points. Although annotations were manually generated on sagittal reconstructions, labels were subsequently exported as volumetric NIFTI masks for all experimental configurations. Nevertheless, because annotation continuity was defined primarily in the sagittal plane, the resulting volumetric representation may have inherently favored sagittal-plane training compared with fully volumetric approaches.

Model development was based on a convolutional neural network architecture derived from the U-Net framework. The final implementation used nnU-Net, which automatically configures preprocessing, architecture, and training parameters [12]. Model training and inference were performed using Python version 3.12.8 and PyTorch version 2.3.1, on a workstation equipped with an NVIDIA GeForce RTX 3060 GPU with 12 GB VRAM.

Five-fold cross-validation was applied. In each fold, 79 patients were used for training and 20 for validation, with rotation of subsets across folds. Training was performed for a minimum of 500 epochs with early stopping criteria.

Four experimental configurations were evaluated: multi-class 2D segmentation, binary 2D segmentation, binary 3D segmentation, and sagittal-plane binary segmentation. Performance was assessed using the Dice similarity coefficient (DSC), precision, recall, and Hausdorff distance on the independent test dataset. For internal evaluation, the maximum Hausdorff distance was reported, whereas for external validation, the 95th percentile Hausdorff distance (HD95) was used to reduce sensitivity to outliers.

The first experimental configuration consisted of a four-class segmentation setup (three positive classes and background) while preserving the original class distribution and image characteristics.

Due to the limited performance of the initial multi-class configuration, the second experiment (2D axial—binary labels) considered all 3 classes as being ‘positive’, while the background was ‘negative’. Additionally, the class weights were added for training, considering a 0.01 weight for the background and a 1.0 weight for the label. This approach resulted in improved segmentation performance compared with the initial multi-class configuration; however, the accuracy remained insufficient for reliable clinical application.

The third experiment explored volumetric 3D segmentation to incorporate additional spatial contextual information that could potentially improve model performance. The labels were the same as in the second experiment, while reformulating the task as a binary segmentation problem. Training time was substantially longer and did not result in improved segmentation performance.

Next, the fourth experiment (2D binary labels—sagittal images) evaluated sagittal-plane iteration instead of axial-plane iteration, as annotations had originally been performed on sagittal slices. This approach yielded the best performance among all evaluated configurations. The rationale for sagittal-plane training emerged after observing that predictions generated from axial-plane training exhibited stepwise discontinuities on sagittal reconstructions, whereas the reference annotations demonstrated smooth, continuous contours. This suggested a mismatch between the annotation geometry and the model’s input representation.

External validation was performed on an independent dataset of 10 CBCT scans obtained from a separate private clinic not involved in model development. Acquisition parameters were not standardized and differed from those of the training dataset, reflecting variations in clinical imaging protocols.

## 3. Results

### 3.1. Anatomical Characteristics of the MLF

All 106 patients (55 females, 51 males) presented at least one MLF, corresponding to a prevalence of 100% within the study sample. Participants’ ages ranged from 18 to 73 years (mean 39.8 ± 19.3 years).

A total of 144 MLF were identified. Most patients (63%) presented a single MLF, whereas 30% and 7% showed double and triple canals, respectively. The majority of canals (72%) exhibited a descending direction, while only 2% showed a horizontal course.

Mean LH was 0.57 ± 0.21 mm, mean LL was 8.61 ± 2.37 mm, and mean LW was 0.70 ± 0.22 mm. Mean distances were as follows: SDB 21.38 ± 4.04 mm, SDL 18.30 ± 5.30 mm, IDB 6.51 ± 1.54 mm, IDL 9.21 ± 4.56 mm, and DB 5.29 ± 1.29 mm.

Interobserver reliability analysis demonstrated excellent agreement. The ICC for morphometric measurements was 0.97 (95% CI: 0.94–0.99).

### 3.2. AI-Based Analysis

The dataset included 49,619 image slices derived from 106 CBCT volumes. An independent test cohort comprising seven patients (3472 slices) was reserved for evaluation. All scans exhibited consistent voxel spacing and an equal number of slices across axial, sagittal, and coronal planes.

The results for the evaluated models are summarized in Table 1. The multi-class segmentation approach showed limited performance, achieving a Dice coefficient of 0.36 on the test set. The binary 2D model demonstrated moderate improvement, with validation and test Dice scores of 0.53 and 0.45, respectively. A similar trend was observed for the binary 3D configuration, which yielded validation and test Dice scores of 0.56 and 0.42, respectively.

**Figure 3:**
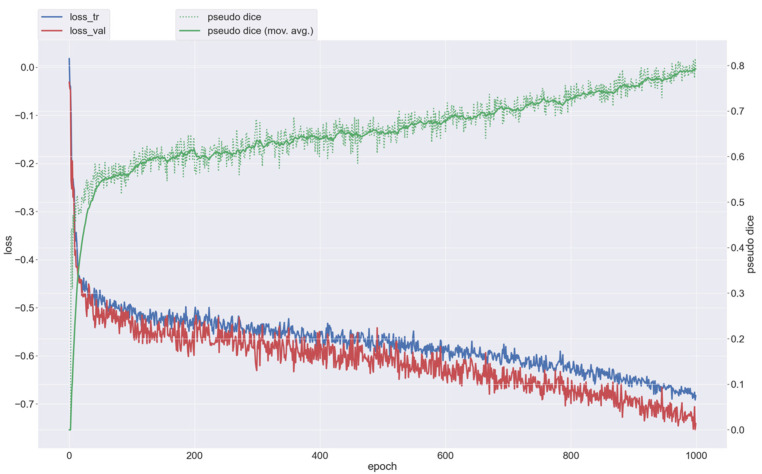
Performance metrics for the sagittal-plane binary segmentation model.

The best results were obtained with the sagittal-plane binary segmentation model, which achieved a validation Dice score of 0.80 and a test Dice score of 0.79. This model also showed good precision (0.88) and a recall of 0.73, with a maximum Hausdorff distance of 16.6 mm (Figure 3).

In the external validation dataset, the model maintained comparable performance, with a mean DSC of 0.81. Detection sensitivity (recall) was 0.71, and precision reached 0.89. The 95th percentile Hausdorff distance was 2.6 mm, while the mean center-point localization error was 1.2 mm, indicating close spatial agreement between automated predictions and reference annotations. At the case level, the medial lingual foramen was correctly identified in 9 out of 10 scans (90%), with the single missed case corresponding to a canal of minimal diameter.

## 4. Discussion

The present study aimed to characterize the anatomical features of the MLF using CBCT imaging and to develop a deep learning-based approach for its automated detection and segmentation. Both objectives were achieved. The morphometric analysis confirmed the consistent presence of the MLF, while also highlighting its anatomical variability. At the same time, the AI-based framework demonstrated that automated segmentation of this small structure is feasible, with the best performance obtained using sagittal-plane input.

The identification of the MLF in all cases supports its frequent anatomical presence within the studied cohort and highlights its potential clinical relevance in the anterior mandible [13]. However, the observed variability in number, size, and spatial distribution highlights the challenges associated with accurate preoperative risk assessment [14]. Even minor differences in foramen morphology may have important implications for hemorrhagic risk, particularly in procedures such as implant placement and bone graft harvesting [15,16].

Although AI has been widely applied in dental imaging [17,18,19,20,21,22,23,24,25,26,27,28], most existing approaches have focused on larger anatomical structures or broader planning tasks [29,30,31,32,33,34,35,36,37]. Previous deep learning studies have reported high performance in these contexts, largely due to the more favorable volumetric representation and contrast of such structures [9,38]. In contrast, the MLF represents a small, low-volume anatomical feature that poses distinct challenges for automated detection. The relatively modest performance of conventional approaches in this study reflects these inherent limitations and highlights the need for task-specific strategies.

Reported Dice scores in studies involving larger anatomical structures, such as the mandibular canal or maxillary sinus, are frequently higher than those obtained in the present study. However, direct comparison remains challenging because segmentation performance is strongly influenced by target size, contrast, anatomical complexity, and volumetric representation. In the case of the MLF, the extremely limited anatomical volume and subtle radiographic appearance represent substantially more demanding segmentation conditions.

In the present study, conventional multi-class formulations and both axial and fully volumetric approaches yielded limited performance, indicating that increasing architectural complexity alone is insufficient to mitigate the effects of extreme class imbalance. This limitation is primarily driven by the minute volumetric representation of the MLF, which accounts for a negligible proportion of the overall image space. Reframing the task as binary segmentation and applying asymmetric class weighting improved detection performance, but these modifications alone did not fully resolve the problem. Instead, model performance was most strongly influenced by the relationship between annotation strategy and input representation.

A key finding of this study is that alignment between annotation geometry and model input plays a critical role in small-structure segmentation. The substantial improvement observed with sagittal-plane input indicates that mismatches between the annotation plane and the training data representation can significantly affect model performance. This highlights an important, yet often overlooked, consideration in the design of deep learning pipelines for anatomically small targets.

Although 3D architectures were explored to incorporate volumetric context, they did not outperform 2D approaches in this setting. This likely reflects not only sensitivity to dataset size and preprocessing constraints but also the mismatch between sagittal-plane annotation geometry and fully volumetric model representation. Because annotation continuity was primarily defined within sagittal reconstructions, volumetric models may have received less spatially consistent supervisory information compared with sagittal-plane training approaches. Overall, these findings suggest that, in data-limited scenarios, careful adaptation of the modeling strategy to the specific task may be more important than increasing architectural complexity. Future research will focus on the development of a dedicated classification model for differentiating single, double, and triple MLF configurations. Given the availability of a functional segmentation framework, classification could be performed directly on the predicted segmented regions rather than on the complete CBCT volume. Additional investigations will explore alternative convolutional neural network architectures and denoising strategies to further improve detection and classification performance. Future studies may also evaluate the integration of device-specific noise modeling derived from phantom imaging in order to improve robustness across different CBCT acquisition systems [39]. Ultimately, the goal is to develop a fully automated pipeline capable of detecting, segmenting, and classifying the MLF in CBCT imaging. From a clinical perspective, the achieved performance is unlikely to replace expert interpretation but may provide meaningful decision support [31]. Although the reported Dice scores remain lower than those typically observed for larger anatomical structures, the achieved localization accuracy may still be clinically meaningful for decision-support applications in implant planning. In the context of very small anatomical targets, overlap-based metrics, such as the Dice similarity coefficient, may demonstrate substantial fluctuations even in the presence of minimal boundary deviations. For this reason, localization-focused metrics may provide a more clinically meaningful assessment of model behavior in small-structure segmentation tasks. The low center-point localization error suggests that the model can reliably identify the approximate anatomical risk region, even in cases where boundary segmentation remains imperfect. The relatively elevated maximum Hausdorff distance was primarily influenced by isolated outlier cases involving fragmented predictions, whereas the low center-point localization error indicated preserved overall localization accuracy. Nevertheless, expert radiological interpretation remains essential, particularly in anatomically complex or low-contrast cases. The proposed framework is not intended to function as an autonomous surgical planning system and should be interpreted exclusively as a decision-support and localization-assistance tool. Such assistance may help improve consistency in CBCT interpretation and reduce the likelihood of overlooking small but clinically relevant structures, particularly in cases where visualization is subtle and operator dependent. Integration of automated MLF detection into routine CBCT workflows may assist clinicians during implant planning by facilitating rapid identification of anatomically high-risk regions. The slightly lower recall observed in the external dataset may reflect differences in image quality or canal morphology, particularly in cases with smaller or less distinct foramina. Despite this, the model maintained relatively consistent overall performance, suggesting preliminary robustness across heterogeneous imaging datasets.

Although the external validation results were encouraging, the relatively small size of the independent cohort limits the robustness of conclusions regarding model generalizability. In addition, variations in acquisition protocols, scanner characteristics, and image quality across institutions may substantially affect model performance. Therefore, larger multicenter validation studies involving heterogeneous CBCT datasets will be necessary before clinical implementation can be considered.

Several limitations should be considered. The relatively limited sample size, particularly for external validation, constrains the robustness of the findings. In addition, the lack of multi-center data prevents a comprehensive assessment of performance across different imaging systems and acquisition protocols. Future studies should focus on larger, more diverse datasets and prospective validation to establish clinical utility. An additional limitation is that intraobserver variability was not formally evaluated, which may influence the reproducibility of manual annotations. Because manual expert annotations served as the reference standard, variability in human interpretation may have influenced both model training and performance evaluation, particularly considering the anatomical variability and small dimensions of the MLF.

In summary, this study demonstrates that deep learning-based segmentation of the MLF is feasible, despite the technical challenges associated with its small size and low volumetric representation. Beyond the specific application, the findings highlight the importance of aligning the annotation strategy with the model’s input representation, providing a generalizable insight for small-structure segmentation tasks. This approach may contribute to more standardized and reproducible preoperative assessment in implant dentistry.

## 5. Conclusions

CBCT remains an essential imaging modality for the accurate identification of the medial lingual foramen, a structure with direct relevance for surgical safety in the anterior mandible. The results of this study indicate that deep learning-based detection and segmentation of the MLF is feasible and may support automated localization assistance during preoperative assessment. Reliable localization of this structure may facilitate identification of anatomically relevant vascular regions during preoperative implant assessment. These findings also suggest that alignment between annotation strategy and model input is an important factor in the segmentation of small anatomical structures. Although the present findings demonstrate the feasibility of AI-assisted MLF segmentation, further multicenter validation on larger and more heterogeneous datasets is required before integration into routine clinical workflows can be recommended.

## Figures and Tables

**Table 1:** Performance comparison of the four experimental configurations.

Experiment	Input	Labels	Plane	Dice Score
1st	2D	Multiclass	Axial	0.36
2nd	2D	Binary	Axial	0.53
3rd	3D	Binary	Volumetric	0.56
4th	2D	Binary	Sagittal	0.79

## Data Availability

The datasets generated and/or analyzed during the current study are available from the corresponding author upon reasonable request, subject to institutional and ethical restrictions.

## Abbreviations

The following abbreviations are used in this manuscript:

MLF	Medial lingual foramen
CBCT	Cone-beam computed tomography
AI	Artificial intelligence
kV	Kilovoltage
mA	Milliamperage
s	Seconds
FOV	Field of view
mm	Millimeters
LH	Canal height
LW	Canal width
LL	Canal length
SDB	Distance superior buccal
SDL	Distance superior lingual
IDB	Distance inferior buccal
IDL	Distance inferior lingual
DB	Deepest buccal distance of the MLF
DICOM	Digital Imaging and Communications in Medicine
NIFTI	Neuroimaging Informatics Technology Initiative
ICC	Intraclass correlation coefficient
DSC	Dice Similarity Coefficient
